# Supplementary material for: Molecular beam homoepitaxy of N-polar AlN: Enabling role of aluminum-assisted surface cleaning
Source: Sci Adv. 2022 Sep 9;8(36):eabo6408. doi: 10.1126/sciadv.abo6408 (PMC9462693; doi:10.1126/sciadv.abo6408)
Supplement: Supplementary file 1 — Supplementary Text Figs. S1 and S2 References [file sciadv.abo6408_sm.pdf]

Science Advances
AAAS

Supplementary Materials for

Molecular beam homoepitaxy of N-polar AlN: Enabling role of aluminum-assisted surface cleaning

Zexuan Zhang *et al.*

Corresponding author: Zexuan Zhang, zz523@cornell.edu; YongJin Cho, yongjin.cho@cornell.edu

Sci. Adv. **8**, eabo6408 (2022)
DOI: 10.1126/sciadv.abo6408

This PDF file includes:

Supplementary Text
Figs. S1 and S2
References

Supplementary Text

Microstructures of the defects in AlN samples were studied using cross-sectional (S)TEM. The actual nature of the defects was identified using cross-sectional TEM (Fig. S1), where the Burgers vector ***b*** of threading dislocations are determined by adjusting the diffraction vector g using the $\boldsymbol{g}\cdot\boldsymbol{b}$=0 criterion. Non centrosymmetric reflection ***g***=0002 and 000$\bar{2}$ were also used to detect any inversion domains which appear as contrast inversion in images taken with these diffraction vectors (*32*).

Temperature-dependent Hall-effect measurements reveal the presence of high-density polarization-induced two-dimensional electron gas (2DEG) at a N-polar $GaN/Al_{0.9}Ga_{0.1}N$ heterostructure (Fig. S2A) grown on N-polar AlN template after Al-assisted surface cleaning. The measured electron density of ~ 3.6×10^{13} cm^{-2} (Fig. S2C), which maintains down to 10 K, indicates its polarization-induced origin, and agrees with the calculated value of 4.2×10^{13} cm^{-2} based on a self-consistent 1D Schrödinger-Poisson simulation shown in Fig. S2B. Figure S2D shows the electron mobility as a function of temperature. The electron mobility increases monotonically with decreasing temperature from 190 $cm^2V^{-1}s^{-1}$ at 290 K to 240 $cm^2V^{-1}s^{-1}$ at 10 K, due to freeze-out of phonon scattering. On the other hand, nominally the same $GaN/Al_{0.9}Ga_{0.1}N$ heterostructure grown on N-polar AlN template without Al-assisted cleaning shows insulating behavior, indicating the absence of such a polarization-induced 2DEG.

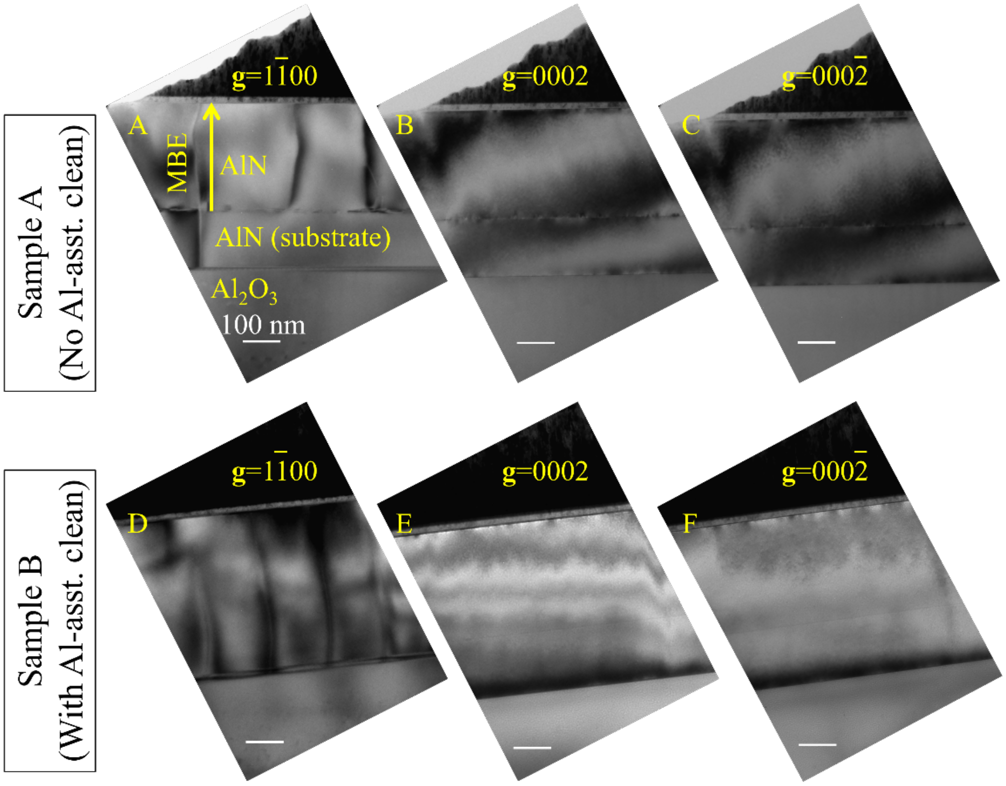

Fig. S1. Two-beam bright-field cross-sectional TEM images of AlN grown on AlN templates. (A to C) Sample A and (D to F) sample B near the <11$\bar{2}$0> zone axis with *g*=1$\bar{1}$00 (A and D), *g*=0002 (B and E) and *g*=000$\bar{2}$ (C and F).

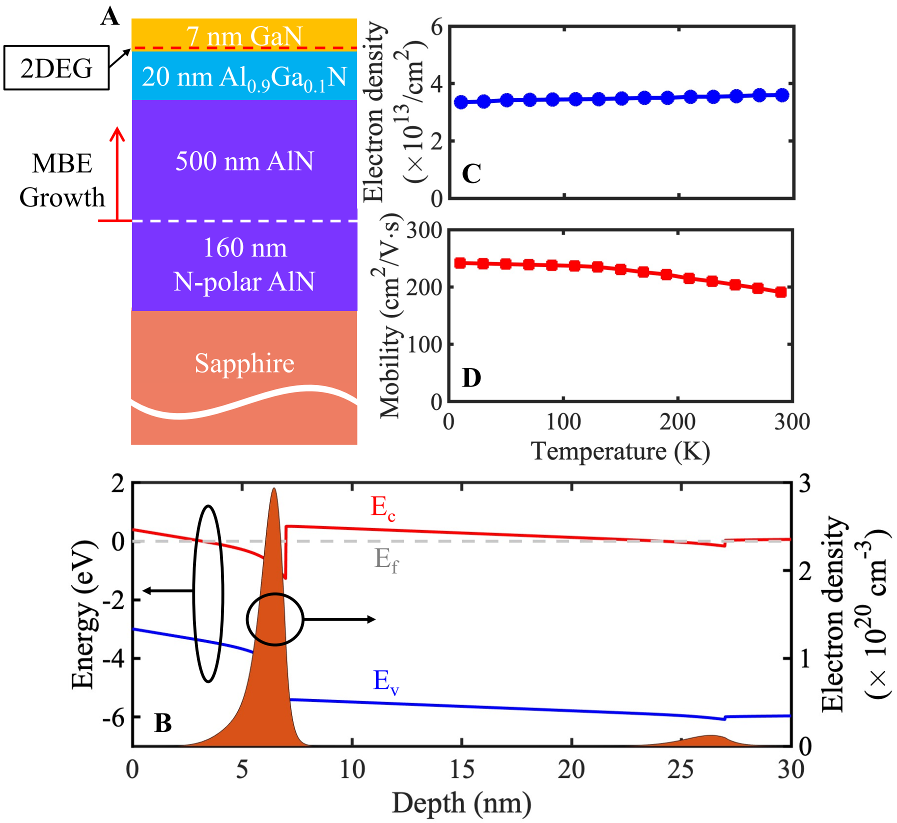

Fig. S2. $GaN/Al_{0.9}Ga_{0.1}N$ heterostructure grown on N-polar AlN template cleaned by Al-assisted deoxidation. (A) Schematic and (B) simulated energy band diagram of the structure. (C) Electron density and (D) Hall mobility as a function of temperature.

REFERENCES AND NOTES

1. A. L. Hickman, R. Chaudhuri, S. J. Bader, K. Nomoto, L. Li, J. C. M. Hwang, H. Grace Xing, D. Jena, Next generation electronics on the ultrawide-bandgap aluminum nitride platform. *Semicond. Sci. Technol.* **36**, 044001 (2021).

2. B. Romanczyk, X. Zheng, M. Guidry, H. Li, N. Hatui, C. Wurm, A. Krishna, E. Ahmadi, S. Keller, U. K. Mishra, W-band power performance of SiN-passivated N-polar GaN deep recess HEMTs. *IEEE Electron Device Lett.* **41**, 349–352 (2020).

3. K. H. Hamza, D. Nirmal, A review of GaN HEMT broadband power amplifiers. *Int. J. Electron. Commun.* **116**, 153040 (2020).

4. M. H. Wong, Y. Pei, T. Palacios, L. Shen, A. Chakraborty, L. S. McCarthy, S. Keller, S. P. DenBaars, J. S. Speck, U. K. Mishra, Low nonalloyed Ohmic contact resistance to nitride high electron mobility transistors using N-face growth. *Appl. Phys. Lett.* **91**, 232103 (2007).

5. M. H. Wong, M. H. Wong, Y. Pei, R. Chu, S. Rajan, B. L. Swenson, D. F. Brown, S. Keller, S. P. Den Baars, J. S. Speck, U. K. Mishra, N-face metal-insulator-semiconductor high-electron-mobility transistors with AlN back-barrier. *IEEE Electron Device Lett.* **29**, 1101–1104 (2008).

6. J. Lemettinen, H. Okumura, T. Palacios, S. Suihkonen, N-polar AlN buffer growth by metal-organic vapor phase epitaxy for transistor applications. *Appl. Phys. Express* **11**, 101002 (2018).

7. T. Ito, R. Sakamoto, T. Isono, Y. Yao, Y. Ishikawa, N. Okada, K. Tadatomo, Growth and characterization of nitrogen-polar AlGaN/AlN heterostructure for high-electron-mobility transistor. *Phys. Status Solidi B* **257**, 1900589 (2020).

8. A. Hickman, R. Chaudhuri, S. J. Bader, K. Nomoto, K. Lee, H. G. Xing, D. Jena , High breakdown voltage in RF AlN/GaN/AlN quantum well HEMTs. *IEEE Electron Device Lett.* **40**, 1293–1296 (2019).

9. J. Lemettinen, N. Chowdhury, H. Okumura, I. Kim, S. Suihkonen, T. Palacios, Nitrogen-polar polarization-doped field-effect transistor based on $Al_{0.8}Ga_{0.2}N$/AlN on SiC with drain current over 100 mA/mm. *IEEE Electron Device Lett.* **40**, 1245–1248 (2019).

10. I. Smorchkova, L. Chen, T. Mates, L. Shen, S. Heikman, B. Moran, S. Keller, S. P. Den Baars, J. S. Speck, U. K. Mishra, AlN/GaN and (Al,Ga)N/AlN/GaN two-dimensional electron gas structures grown by plasma-assisted molecular-beam epitaxy. *J. Appl. Phys.* **90**, 5196 (2001).

11. S. Dasgupta, F. Wu, J. Speck, U. Mishra, Growth of high quality N-polar AlN ($000\bar{1}$) on Si (111) by plasma assisted molecular beam epitaxy. *Appl. Phys. Lett.* **94**, 151906 (2009).

12. O. Ledyaev, M. Pandikunta, S. Nikishin, N-polar AlN thin layers grown on Si (111) by plasma-assisted MBE. *Jpn. J. Appl. Phys.* **53**, 050306 (2014).

13. J. Lemettinen, H. Okumura, I. Kim, C. Kauppinen, T. Palacios, S. Suihkonen, MOVPE growth of N-polar AlN on 4H-SiC: Effect of substrate miscut on layer quality. *J. Cryst. Growth* **487**, 12–16 (2018).

14. T. Isono, T. Ito, R. Sakamoto, Y. Yao, Y. Ishikawa, N. Okada, K. Tadatomo, Growth of N-polar aluminum nitride on vicinal sapphire substrates and aluminum nitride bulk substrates. *Phys. Status Solidi B* **257**, 1900588 (2020).

15. H. Okumura, T. Kimoto, J. Suda, Growth of nitrogen-polar 2H-AlN on step-height-controlled 6H-SiC ($000\bar{1}$) substrate by molecular-beam epitaxy. *Jpn. J. Appl. Phys.* **51**, 02BH02 (2012).

16. K. Shojiki, K. Uesugi, S. Kuboya, H. Miyake, Reduction of threading dislocation densities of N-polar face-to-face annealed sputtered AlN on sapphire. *J. Cryst. Growth* **574**, 126309 (2021).

17. Z. Liu, Y. Guo, J. Yan, Y. Zeng, J. Wang, J. Li, Polarity tuning of crystalline AlN films utilizing trace oxygen involved sputtering and post-high-temperature annealing. *Appl. Phys. Express* **14**, 085501 (2021).

18. S. Keller, N. Fichtenbaum, F. Wu, G. Lee, S. P. DenBaars, J. S. Speck, U. K. Mishra , Effect of the nucleation conditions on the polarity of AlN and GaN films grown on C-face 6H-SiC. *Jpn. J. Appl. Phys.* **45**, L322–L325 (2006).

19. Y. Cho, C. S. Chang, K. Lee, M. Gong, K. Nomoto, M. Toita, L. J. Schowalter, D. A. Muller, D. Jena, H. G. Xing, Molecular beam homoepitaxy on bulk AlN enabled by aluminum-assisted surface cleaning. *Appl. Phys. Lett.* **116**, 172106 (2020).

20. K. Lee, Y.-J. Cho, L. J. Schowalter, M. Toita, H. G. Xing, D. Jena, Surface control and MBE growth diagram for homoepitaxy on single-crystal AlN substrates. *Appl. Phys. Lett.* **116**, 262102 (2020).

21. C. Wurm, E. Ahmadi, F. Wu, N. Hatui, S. Keller, J. Speck, U. Mishra , Growth of high-quality N-polar GaN on bulk GaN by plasma-assisted molecular beam epitaxy. *Solid State Commun.* **305**, 113763 (2020).

22. Y. P. Hong, J. H. Park, C. W. Park, H. M. Kim, D. K. Oh, B. G. Choi, S. K. Lee, K. B. Shim, Investigation of defects and surface polarity in AlN and GaN using wet chemical etching technique. *J. Korean Cryst. Growth Cryst. Technol.* **24**, 196–201 (2014).

23. R. Kirste, S. Mita, L. Hussey, M. P. Hoffmann, W. Guo, I. Bryan, Z. Bryan, J. Tweedie, J. Xie, M. Gerhold, R. Collazo, Z. Sitar , Polarity control and growth of lateral polarity structures in AlN. *Appl. Phys. Lett.* **102**, 181913 (2013).

24. L. Lu, Z. Y. Gao, B. Shen, F. J. Xu, S. Huang, Z. L. Miao, Y. Hao, Z. J. Yang, G. Y. Zhang, X. P. Zhang, J. Xu, D. P. Yu , Microstructure and origin of dislocation etch pits in GaN epilayers grown by metal organic chemical vapor deposition. *J. Appl. Phys.* **104**, 123525 (2008).

25. W. Guo, R. Kirste, I. Bryan, Z. Bryan, L. Hussey, P. Reddy, J. Tweedie, R. Collazo, Z. Sitar, KOH based selective wet chemical etching of AlN, $Al_xGa_{1-x}N$, and GaN crystals: A way towards substrate removal in deep ultraviolet-light emitting diode. *Appl. Phys. Lett.* **106**, 082110 (2015).

26. T. Akiyma, M. Uchino, K. Nakamura, T. Ito, S. Xiao, H. Miyake, Structural analysis of polarity inversion boundary in sputtered AlN films annealed under high temperatures. *Jpn. J. Appl. Phys.* **58**, SCCB30 (2019).

27. N. Stolyarchuk, T. Markurt, A. Courville, K. March, J. Zúñiga-Pérez, P. Vennéguès, M. Albrecht , Intentional polarity conversion of AlN epitaxial layers by oxygen. *Sci. Rep.* **8**, 14111 (2018).

28. M. Takeuchi, H. Shimizu, R. Kajitani, K. Kawasaki, T. Kinoshita, K. Takada, H. Murakami, Y. Kumagai, A. Koukitu, T. Koyama, S. F. Chichibu, Y. Aoyagi , Al- and N-polar AlN layers grown on c-plane sapphire substrates by modified flow-modulation MOCVD. *J. Cryst. Growth* **305**, 360–365 (2007).

29. H. Ye, G. Chen, Y. Zhu, S.-H. Wei, Asymmetry of adsorption of oxygen at wurtzite AlN (0001) and $(000\bar{1})$ surfaces: First-principles calculations. *Phys. Rev. B* **77**, 033302 (2008).

30. J. Li, K. Nam, M. Nakarmi, J. Lin, H. Jiang, Band-edge photoluminescence of AlN epilayers. *Appl. Phys. Lett.* **81**, 3365–3367 (2002).

31. M. Feneberg, R. A. Leute, B. Neuschl, K. Thonke, M. Bickermann, High-excitation and high-resolution photoluminescence spectra of bulk AlN. *Phys. Rev. B* **82**, 075208 (2010).

32. L. Romano, J. Northrup, M. O'keefe, Inversion domains in GaN grown on sapphire. *Appl. Phys. Lett.* **69**, 2394–2396 (1996).
